# Co-administration of HAART and antikoch triggers cardiometabolic dysfunction through an oxidative stress-mediated pathway

**DOI:** 10.1186/s12944-021-01493-x

**Published:** 2021-07-05

**Authors:** RE Akhigbe, MA Hamed

**Affiliations:** 1 Department of Physiology, Ladoke Akintola University of Technology, Ogbomoso, Oyo State Nigeria; 2 Reproductive Biology and Toxicology Research Laboratories, Oasis of Grace Hospital, Osogbo, Osun State Nigeria; 3 Department of Chemical Sciences, Kings University, Odeomu, Osun Nigeria; 4 Buntai Medical and Diagnostic Laboratories, Osogbo, Nigeria

**Keywords:** Anti-tuberculosis, Anti-retroviral, Oxidative stress, Metabolic disorder, Inflammation, Apoptosis

## Abstract

**Background:**

Antikoch and highly active anti-retroviral therapy are effective drugs in the management of tuberculosis and Human Immunodeficiency Virus, respectively. However, these cocktails have been independently associated with the aetiopathogenesis of metabolic syndrome. This study investigated whether or not the co-administration of antikoch and anti-retroviral, as seen in tuberculosis/Human Immunodeficiency Virus co-infection, will produce a similar effect. Also, it evaluated the role of glutathione and adenine deaminase/xanthine oxidase/uric acid signaling in antikoch/anti-retroviral-induced cardiometabolic dysfunction.

**Methods:**

Male rats of Wistar strain were randomized into four groups: the control, which had 0.5 mL of distilled water as a vehicle, anti-Koch-treated rats that were administered a cocktail of anti-Koch, HAART-treated rats that had a combination of anti-retroviral drugs, and anti-Koch + HAART-treated rats that had treatments as anti-Koch-treated and HAART-treated rats. The treatment was once daily and lasted for eight weeks. One way-analysis of variance followed by Tukey’s posthoc test was used to test for significance and pairwise comparisons respectively.

**Results:**

Although no changes in body weight gain and cardiac weight were noted, it was found that antikoch and/or HAART caused insulin resistance and elevated blood glucose level. In addition, antikoch and/or HAART led to dyslipidaemia, increased atherogenic indices, and elevated cardiac injury markers. These were accompanied by increased plasma and cardiac concentrations of malondialdehyde and nitric oxide, C-reactive protein, and myeloperoxidase activity, as well as suppressed activities of glutathione peroxidase and glutathione-S-transferase, and a fall in reduced glutathione level. The observed alterations were more pronounced in animals that received a combination of antikoch and HAART.

**Conclusions:**

This study provides the first evidence that antikoch and/or HAART induce cardiometabolic dysfunction via glutathione suppression and up-regulation of adenine deaminase/xanthine oxidase/uric acid-dependent oxidative stress and inflammatory response. These events were associated with dyslipidaemia and increased atherogenic indices. This infers that regular monitoring of glucose level, insulin sensitivity, lipid profile, and oxido-inflammatory markers is important in patients on antikoch and/or HAART for prompt diagnosis and management of cardiometabolic disorder if it ensues.

## Introduction

Despite the improved management of infectious diseases, Human Immunodeficiency Virus-tuberculosis (HIV-TB) co-infection remains a major health challenge globally. About 2 billion of the global population is latently infected with TB [1], while 35.3 million people live with HIV [2] with preponderance in sub-Saharan Africa [2]. HIV has been the leading cause of death followed by TB, with an estimated global annual incidence of 2.5 million and 8.7 million, respectively [1, 2]. About 12–13 % of TB-infected individuals are co-infected with HIV [1, 3]. The established epidemiological and biological links between these infectious diseases influence the disease’s spread, progression, and prognosis. HIV is a predisposing factor for incident TB. It is also a major factor in the resurgence of TB. HIV-induced immunosuppression increases the susceptibility of HIV-infected individuals to infections such as TB. HIV modifies the development of TB and increases the risk of incident active TB in people with latent TB [1]. The increased incidence of HIV-TB co-infection has led to increased concomitant use of highly active anti-retroviral therapy (HAART) and anti-tuberculosis (antikoch). Although these drugs have been shown to improve the clinical status of infected patients significantly, studies have reported that they also induce hepatorenal toxicity [4–6] and possibly cardiometabolic dysfunction and cardiovascular complications [7–11].

Though there are scanty reports on the impact of TB and antikoch on cardiometabolic disorder, Thanoon and Alrahman [7] observed that antikoch but not active TB caused a significant rise in body mass index (BMI), serum leptin, and triglyceride. Interestingly, most studies on TB and metabolic disorder linked active TB infection rather than antikoch, with a rise in incident cardiometabolic disorder. Globally, 70 % of people with diabetes live in TB endemic countries [12]. Kibirige et al. [13] demonstrated that diabetes mellitus is found in a large number of hospitalized patients with TB. Niazi and Kalra [14], in their study reported the bi-directional relationship between diabetes and TB. They showed that diabetes is a non-reliant predisposing factor for infections of the lower respiratory tract [14, 15], including TB. TB has also been shown to cause tuberculous pancreatitis [16] with an attendant hyperglycaemic state. In non-diabetics, rifampicin enhances intestinal glucose absorption with resultant hyperglycaemia [17].

Similarly, a handful of studies implicated HAART use in the pathogenesis of cardiometabolic disorders. Human studies have reported that exposure to protease and nucleoside reverse transcriptase inhibitors induce metabolic syndrome and endothelial dysfunction [10, 18–22]. Recently, it was reported that integrase inhibitors triggered dyslipidaemia [23]. HAART-induced cardiometabolic dysfunction is via depletion of leptin and adiponectin, resulting in insulin resistance, dyslipidaemia, hyper-triglycaemia, and lowered high-density lipoprotein (HDL) [8, 24]. In addition, Murata et al. [25] demonstrated that protease inhibitors selectively suppress glucose transporter 4 (GLUT4) activity, hence impairing glucose transport into the cells leading to hyperglycemia. HAART-induced cardiometabolic dysfunction has also been attributed to anti-retroviral-led mitochondrial dysfunction and elevation of serum lactate [26]. Despite the findings mentioned above, the role of glutathione and adenine deaminase (ADA)/xanthine oxidase (XO)/uric acid (UA) signaling in HAART-/antikoch-induced cardiometabolic dysfunction is yet to be elucidated.

Oxidative stress is one of the culprits that trigger cardiometabolic dysfunction. A trigger of oxidative stress is the up-regulation of ADA/XO/UA signaling. Purines, basically adenosine, and guanine, are broken down into hypoxanthine, which is metabolized by xanthine dehydrogenase to xanthine. Xanthine is further degraded into uric acid by xanthine oxidoreductase, which usually exists as xanthine dehydrogenase that can be irreversibly cleaved by proteolysis or reversibly oxidized into xanthine oxidase [27, 28]. UA induces oxidative stress by reacting with peroxynitrite, a product of nitric oxide (NO) reaction with superoxide radical. Hence UA depletes NO bioavailability, promotes inflammation and oxidative stress, and induces insulin resistance, glucose intolerance/hyperglycaemia, dyslipidaemia, and impaired cardiovascular function [28–30]. Besides, ADA irreversibly deaminates adenosine and deoxyadenosine, known antioxidant and anti-inflammatory molecules, into inosine and deoxyinosine [31], thus depleting the oxido-inflammatory defense system, including the glutathione system, and increasing the susceptibility of cells/tissues/organs to oxidative damage.

Given these activities of antikoch and HAART and the knowledge that elevated uric acid is central in the pathogenesis of cardiometabolic syndrome, this study investigated the hypothesis that antikoch and HAART, independently and in synergy, induce cardiometabolic dysfunction possibly by suppressing glutathione and up-regulating ADA/XO/UA signaling.

## Materials and methods

### Animals and chemicals

The present study used 32 in-bred litter-mate adult male rats of Wistar strain and comparable weight. Animals were kept in polypropylene cages (43 cm long × 29.5 cm wide × 24 cm deep) with netted covers (4 rats/ cage) and maintained under natural light/dark cycle. Rats were allowed to feed on normal rat diet and drink water ad’libitum. The data from our pilot study revealed that the impact of HAART and antikoch on cardiometabolic function was not gender-bias. Hence for homogeneity, only male animals were used for this study. The Research Ethics Committee of Oasis of Grace Hospital approved the study protocol (OG/2019/032), and the study was carried out per the guidelines of the “National Institute of Health using the guide for the care and handling of laboratory animals (NIH Publication No. 80 − 23; revised 1978), and International Guiding Principles for Biomedical Research (CIOMS, 1985)”. All reagents used were of analytical grade and procured from Sigma-Aldrich, USA, except otherwise stated.

### Experimental design

Rats were randomized after two weeks of acclimatization into one of the four groups (n = 8); the control, anti-Koch-treated, HAART-treated, and anti-Koch + HAART-treated. The control animals had 0.5 mL of distilled water as a vehicle, anti-Koch-treated rats were administered a combination of anti-Koch (Rifampicin, Isoniazid, Pyrazinamide, and Ethambutol), HAART-treated rats had a combination of anti-retroviral drugs (Efavirenz, Lamivudine, and Tenofovir), while the Anti-Koch + HAART-treated rats had treatments as anti-Koch-treated and HAART-treated rats. Human Equivalent Doses of the drugs for rats “(HAART: 52.9 mg/kg of Efavirenz, 26.48 mg/kg of Lamivudine, and 26.48 mg/kg of Tenofovir; and anti-Koch: 61.66 mg/kg of Rifampicin, 30.83 mg/kg of Isoniazid, 154.15 mg/kg of Pyrazinamide, and 92.49 mg/kg of Ethambutol)” were used [6, 32]. All drugs and vehicle were administered orally using an oropharyngeal cannula. The administration was once daily and lasted for eight weeks as prescribed for TB patients during the intensive phase of TB management. As seen in human antikoch/HAART co-therapy, administration of HAART started two weeks after anti-Koch treatment [6, 32].

### Sample collection

The experiment was terminated after eight weeks by culling the overnight-fasted animals under intraperitoneal ketamine and xylazine anaesthesia at 40 mg/kg and 4 mg/kg respectively as earlier reported [33–35] after weighing. Blood was collected through cardiac puncture from all the rats and collected into heparinized tubes, centrifuged at 3000 g for 5 min to obtain the plasma, which was refrigerated till required for biochemical studies. The heart of each rat was quickly removed, trimmed of adhering structures, blotted, and weighed. The relative cardiac weight was obtained as cardiac weight/final body weight X 100. A homogenate of a fraction of the cardiac tissue was made in an appropriate measure of cold phosphate-buffered solution and centrifuged at 10, 000 g for 10 min at 4 °C to obtain the supernatant for biochemical analysis.

### Biochemical assays

Fasting blood glucose (FBG) concentration, plasma triglycerides (TG), total cholesterol (TC), low-density lipoproteins (LDL-C), very low-density lipoproteins (VLDL), and high-density lipoproteins (HDL-C) were assayed by colorimetric method using standard laboratory reagents (Randox Laboratory Ltd., UK). Plasma non-HDL, a more reliable marker of dyslipidaemia, was determined as the difference between total cholesterol and HDL-C. Atherogenic indices were calculated and expressed as atherogenic index (Log TG/HDL-C) and atherogenic coefficient (TC-HDL-C)/HDL-C) [37]. Triglyceride-glucose index (TyG), an index of insulin resistance (IR), was estimated using Ln (TG (mg/dl) X FBG (mg/dl)/2) [36, 37].

Cardiac creatinine kinase (CK) and lactate dehydrogenase (LDH) activities (Aggappe Diagnostic, Switzerland), troponin (Abbexa, UK), and lactate (Abcam, China) were determined using spectrophotometric method with ELISA kits.

Cardiac and plasma ADA and XO activities and UA concentrations were determined by enzymatic colorimetric assay technique using laboratory kits (Fortress Diagnostic, UK).

Colorimetric methods were employed to assay the cardiac and plasma concentrations of malondialdehyde (MDA) [33, 38], reduced glutathione (GSH) [39], nitric oxide (NO) [33, 39], and activities of superoxide dismutase (SOD) [40, 41], catalase [42], glutathione peroxidase (GPx) [33, 37], glutathione-S-transferase (GST) [33, 38], and myeloperoxidase (MPO) [33].

Cardiac and plasma C-reactive protein (CRP) were assayed using ELISA kit (Creative Diagnostics, USA) per the manufacturer’s guideline.

### Histopathological analysis

A section of the fixed cardiac tissue was dehydrated, embedded in paraffin, and sectioned at 5 μm thickness. Afterwards, hematoxylin and eosin (H&E) stain was applied. The slides were viewed using a light microscope at 100 x and 400 x. Photomicrographs were taken at 400 x. Each photomicrograph is representative of 5 replicates per group.

### Data analysis and statistics

Data are shown as means ± SD. GraphPad Prism for Windows (Versions 5.0) was used for statistical analyses. One-way analysis of variance was employed to assess the differences in the mean values of variables across the groups, and then Tukey’s posthoc test was used for pair-wise comparisons of the mean values among the groups. Statistical significance was set at *P* < 0.05.

## Results

### Antikoch and HAART exposure insignificantly lowered body weight and cardiac weight

Compared with the control, there was a marginal decrease in the body weight gain in antikoch- and HAART exposed rats (Table 1). Concurrent administration of antikoch and HAART led to a further decrease in body weight gain. However, this observation was not statistically significant (Table 1). Similarly, antikoch and HAART, when administered alone or combined, led to insignificant reductions in cardiac weight and relative cardiac weight (Table 1).

**Table 1:** The effect of HAART and antikoch on body weight gain and cardiac weight

	Control	Anti-koch	HAART	Anti-koch + HAART	*P*-value
Body weight gain (%)	26.08 ± 0.77	25.63 ± 0.81	25.63 ± 0.77	25.81 ± 0.65	0.60
Cardiac weight (g)	3.93 ± 0.20	3.70 ± 0.21	3.73 ± 0.22	3.71 ± 0.31	0.68
Relative cardiac weight (g)	2.15 ± 0.12	2.04 ± 0.08	2.08 ± 0.09	2.05 ± 0.09	0.77

Data are presented as mean ± SD of 8 replicates

### HAART impairs glucoregulation

**Fig. 1:**
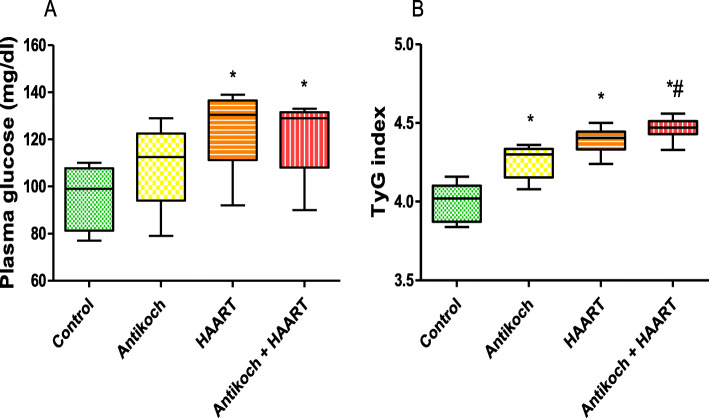
Effects of antikoch and HAART on (**A**) plasma glucose concentration and (**B**) insulin resistance determined by TyG index. TyG means triglyceride-glucose index. It is a measure of insulin resistance and estimated it is. Ln (Triglyceride (mg/dl) X Fasting blood sugar (mg/dl)/2). Data are presented as mean ± SD of 8 replicates; *P < 0.05 vs. control; #*P <* 0.05 vs. antikoch; ~*P <* 0.05 vs. HAART

Fasting glycaemia was comparable between the antikoch-treated and vehicle-treated animals (Fig. 1). HAART administration alone and in combination with antikoch led to a significant increase in fasting blood glucose. In comparison with the control, antikoch or HAART led to significantly increased TyG-index, which was further increased when HAART was concomitantly administered with antikoch (Fig. 1).

### HAART exaggerates antikoch-induced lipid dysregulation

**Fig. 2:**
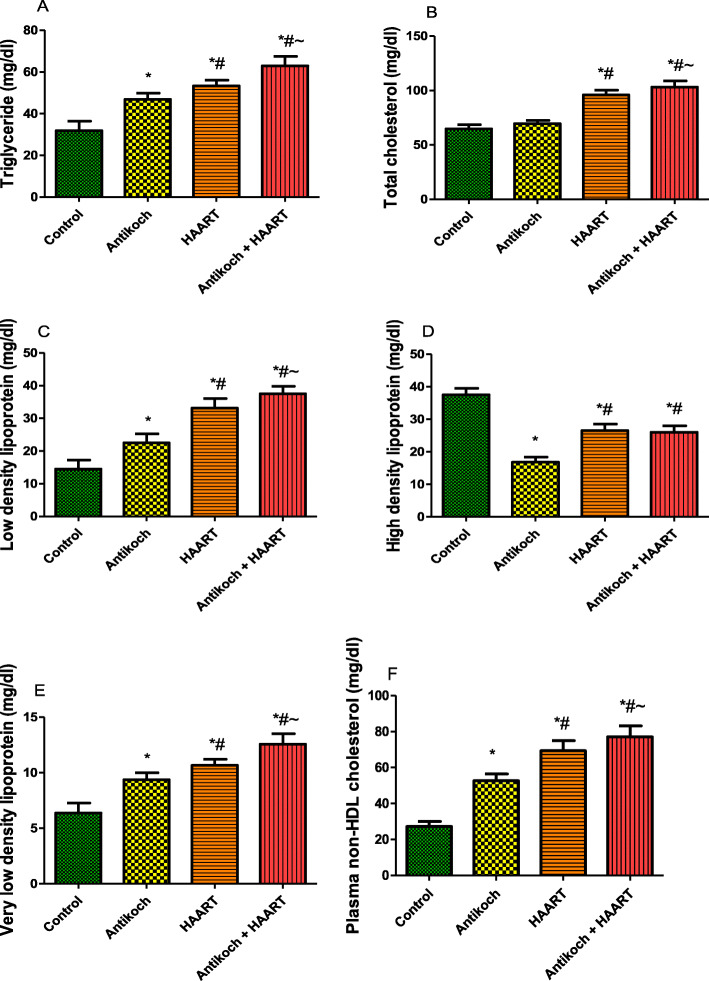
Effects of antikoch and HAART on plasma levels of triglyceride (**A**), total cholesterol (**B**), low density lipoprotein (**C**), high density lipoprotein (**D**), very low density lipoprotein (**E**), and plasma non-HDL cholesterol. Data are presented as mean ± SD of 8 replicates; *P< 0.05 vs control; # *P* < 0.05 vs antikoch; ~ *P* < 0.05 vs HAART

Antikoch treatment or HAART exposure led to a significant rise in plasma TG, LDL, and VLDL compared with the control (Fig. 2). These observations were significantly more in HAART-treated rats when compared with the antikoch-treated rats. Interestingly, concurrent administration of antikoch and HAART led to a more prominent increase in plasma TG, LDL, and VLDL accumulation when compared to all other groups. Although plasma TC was comparable between the antikoch exposed rats and control, HAART caused a significant rise in plasma TC when administered alone and in combination with antikoch. In addition, antikoch exposure and HAART administration led to a significant fall in plasma HDL when compared with the vehicle-treated control. The observed decline in plasma HDL in HAART- and antikoch + HAART- treated rats was significant compared with that in antikoch-treated animals. Furthermore, treatments with antikoch and HAART, singly or concurrently, led to a significant rise in plasma non-HDL cholesterol compared with the control; the noted rise in non-HDL cholesterol was significant in HAART-treated rats when compared with antikoch-treated rats and also significant in animals who received antikoch and HAART when compared with either antikoch-treated or HAART-treated animals (Fig. 2).

### Antikoch and HAART induce atherogenic dyslipidaemia

**Fig. 3:**
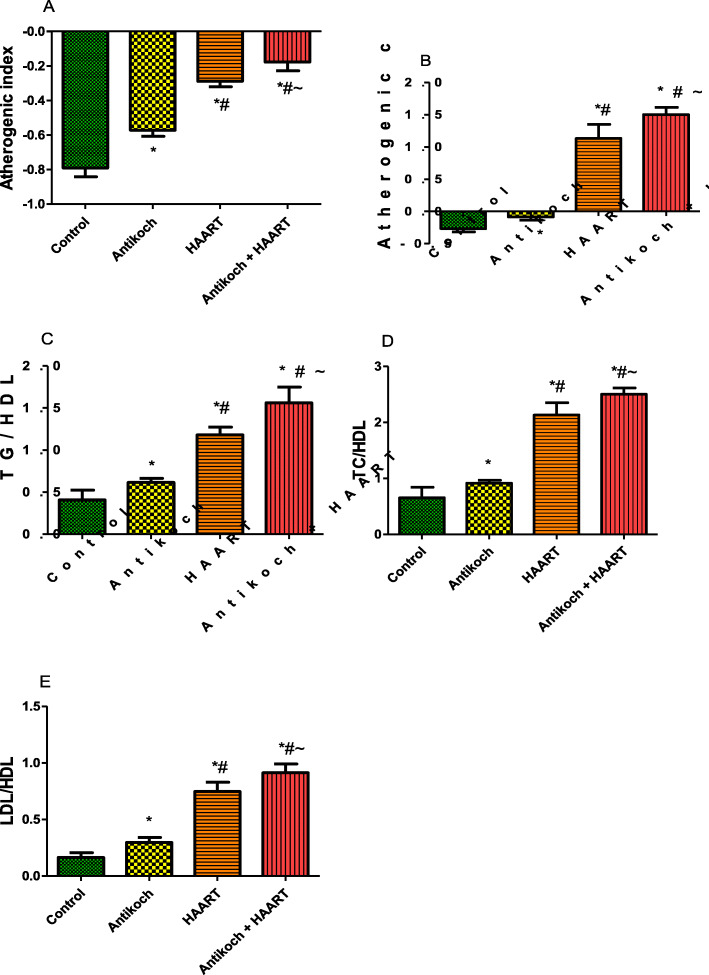
Effects of antikoch and HAART on plasma levels of atherogenic index (**A**), atherogenic coefficient (**B**), TG/HDL (**C**), TC/HDL (**D**), and LDL/HDL (**E**). Atherogenic index and atherogenic coefficient are markers of the risk of atherosclerosis and calculated as (Log TG/HDL-C) and (TC-HDL-C)/HDL-C) respectively. TG: triglyceride; TC: total cholesterol; HDL: high density lipoprotein; LDL: low density lipoprotein. Data are presented as mean ± SD of 8 replicates; * *P* < 0.05 vs control; # *P* < 0.05 vs antikoch; ~ *P* < 0.05 vs HAART

Indices of atherogenic dyslipidaemia such as atherogenic index, atherogenic co-efficient, TG/HDL, TC/HDL, and LDL/HDL, were significantly elevated by antikoch treatment or HAART exposure compared with the control (Fig. 3). HAART exposure led to a more significant rise in these atherogenic variables than antikoch administration. It is noteworthy that the concomitant administration of antikoch and HAART led to a significant increase in these atherogenic indices compared with antikoch or HAART monotherapy (Fig. 3).

### Antikoch and HAART exposure elevates cardiac injury markers

**Fig. 4:**
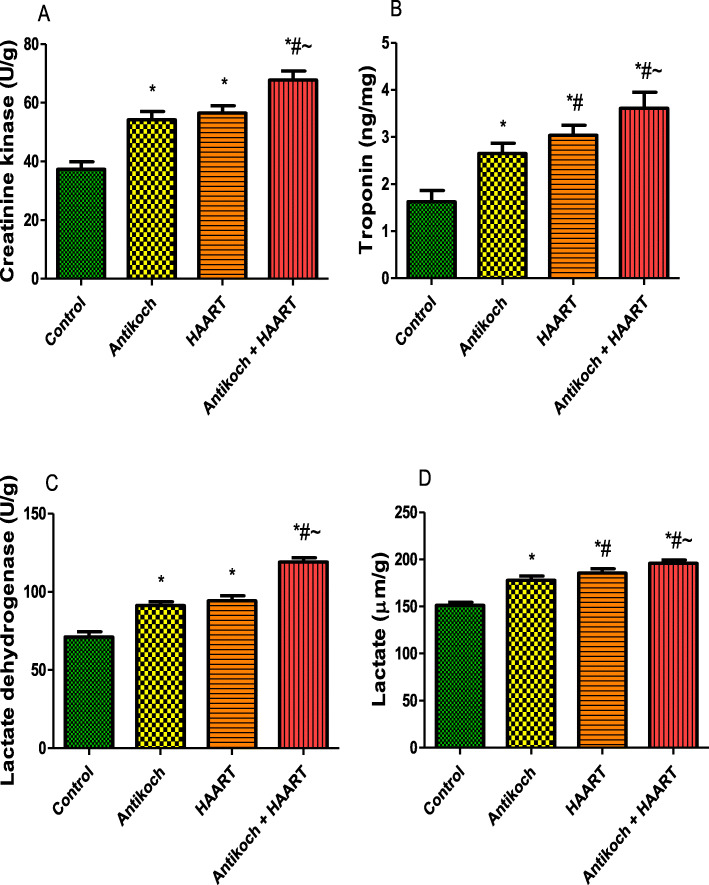
Effects of antikoch and HAART on cardiac creatinine kinase activity (**A**), troponin concentration (**B**), lactate dehydrogenase activity (**C**), and lactate concentration (**D**). Data are presented as mean ± SD of 8 replicates; *P < 0.05 vs. control; #*P <* 0.05 vs. antikoch; ~*P <* 0.05 vs. HAART

Although cardiac CK and LDH activities were similar between the antikoch-treated and HAART-exposed rats, these were significantly higher in the treatment groups when compared with the control (Fig. 4). Co-administration of antikoch and HAART caused a further significant rise in these cardiac function enzymes’ activities compared to other groups. In addition, the treatment groups showed a significant increase in cardiac levels of troponin and lactate compared with the vehicle-treated control. The observed rise in cardiac troponin and lactate contents was more in the HAART-exposed animals than in the antikoch-treated animals, and in the antikoch + HAART-treated animals than in the HAART-treated animals (Fig. 4).

### HAART exacerbates antikoch-induced upregulation of ADA/XO/UA signaling

**Fig. 5:**
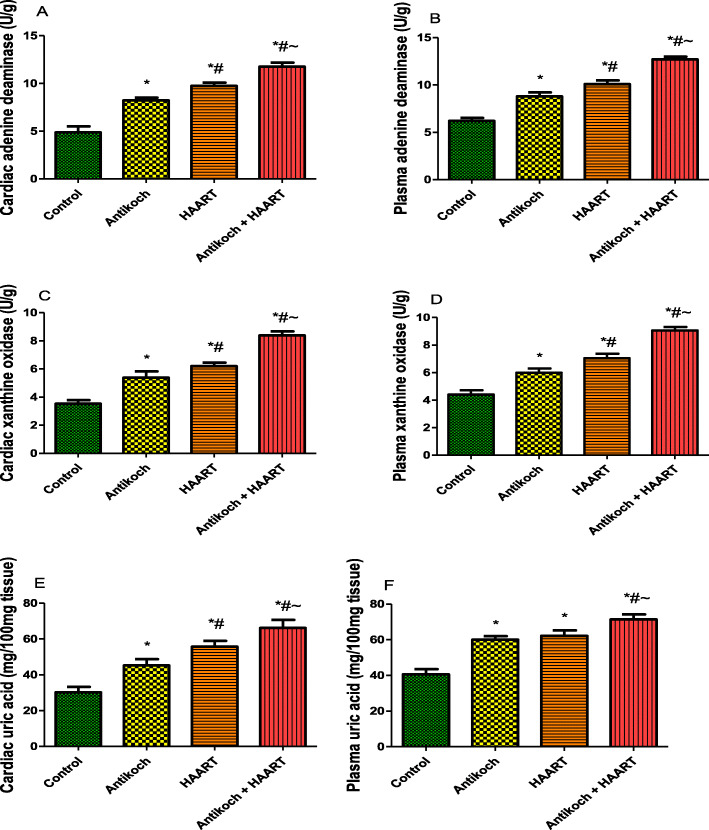
Effects of antikoch and HAART on cardiac and plasma adenine deaminase activities (**A**, **B**) xanthine oxidase activities (**C**, **D**), and uric acid levels (**E**, **F**). Data are presented as mean ± SD of 8 replicates; *P < 0.05 vs. control; #*P <* 0.05 vs. antikoch; ~*P <* 0.05 vs. HAART

Cardiac and systemic ADA activity was significantly increased in antikoch- and HAART-exposed rats when compared with the control (Fig. 5). Co-administration of antikoch and HAART caused a significant rise in ADA activity when compared to all groups. Furthermore, cardiac and plasma XO activity was significantly increased in antikoch-treated and HAART-exposed animals compared with the vehicle-treated control group. Concurrent administration of both cocktails also led to a significant increase in XO activity compared to all groups. Besides, antikoch and HAART treatments, singly and in combination, significantly elevated cardiac and plasma concentrations of UA (Fig. 5).

### Antikoch and HAART treatment induce oxidative stress in heart and blood

**Fig. 6:**
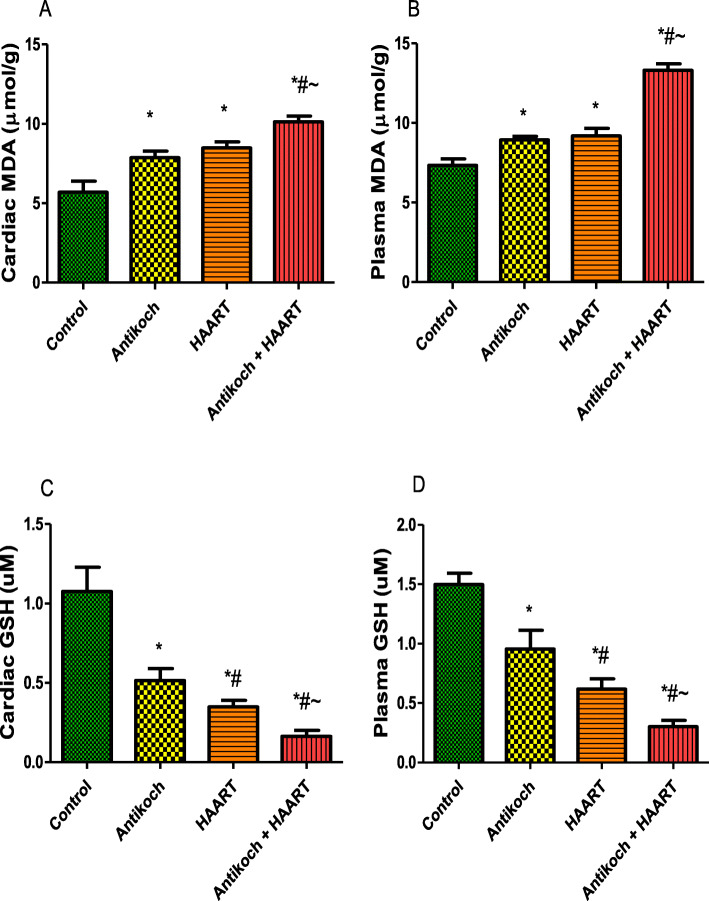
Effects of antikoch and HAART on cardiac and plasma malondialdehyde (MDA) (**A**, **B**), and reduced glutathione (GSH) (**C**,** D**). Data are presented as mean ± SD of 8 replicates; *P < 0.05 vs. control; #*P <* 0.05 vs. antikoch; ~*P <* 0.05 vs. HAART

Antikoch exposure or HAART treatment led to significantly increased cardiac and plasma MDA levels compared with the control group (Fig. 6). However, treatment with either antikoch or HAART significantly reduced cardiac and plasma GSH. The observed alterations in cardiac and plasma MDA and GSH were significantly more in antikoch-HAART co-therapy (Fig. 6).

**Fig. 7:**
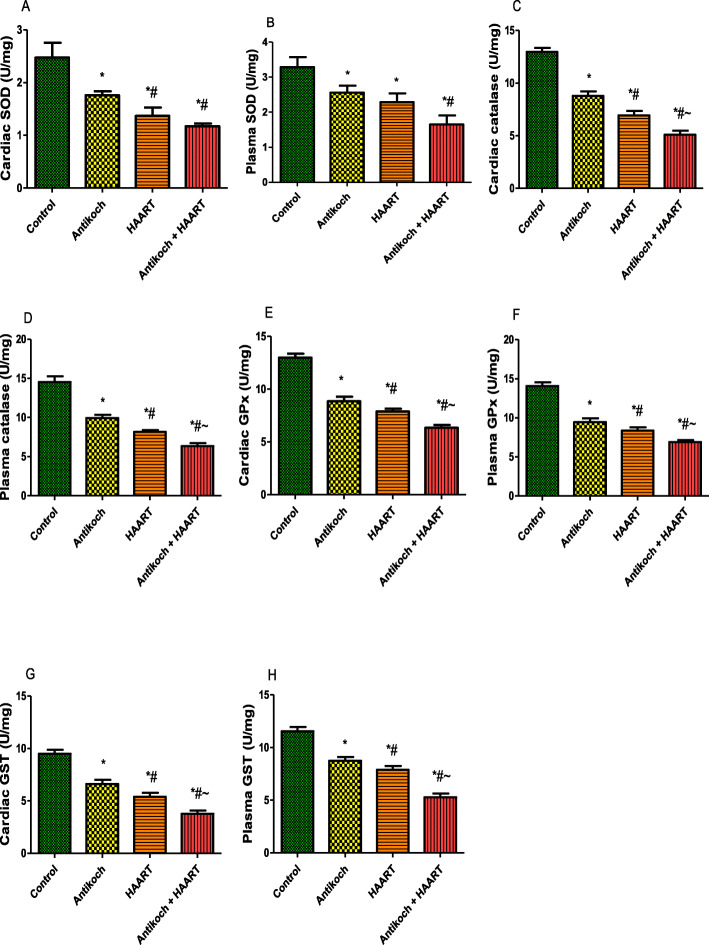
Effects of antikoch and HAART on cardiac and plasma activities of superoxide dismutase (SOD) (**A**, **B**), catalase (**C**, **D**), glutathione peroxidase (**E**, **F**), and glutathione-S-transferase (**G**, **H**). Data are presented as mean ± SD of 8 replicates; * *P* < 0.05 vs control; # *P* < 0.05 vs antikoch; ~ *P* < 0.05 vs HAART

In addition, cardiac and plasma activities of SOD, catalase, GPx, and GST were significantly reduced in antikoch- and HAART-treated rats. Similar to MDA and GSH, the reduction in enzymatic antioxidants activities noted in antikoch- and HAART-exposed animals were significantly more in animals that received both drugs concomitantly (Fig. 7).

### Antikoch and HAART increases inflammatory markers

**Fig. 8:**
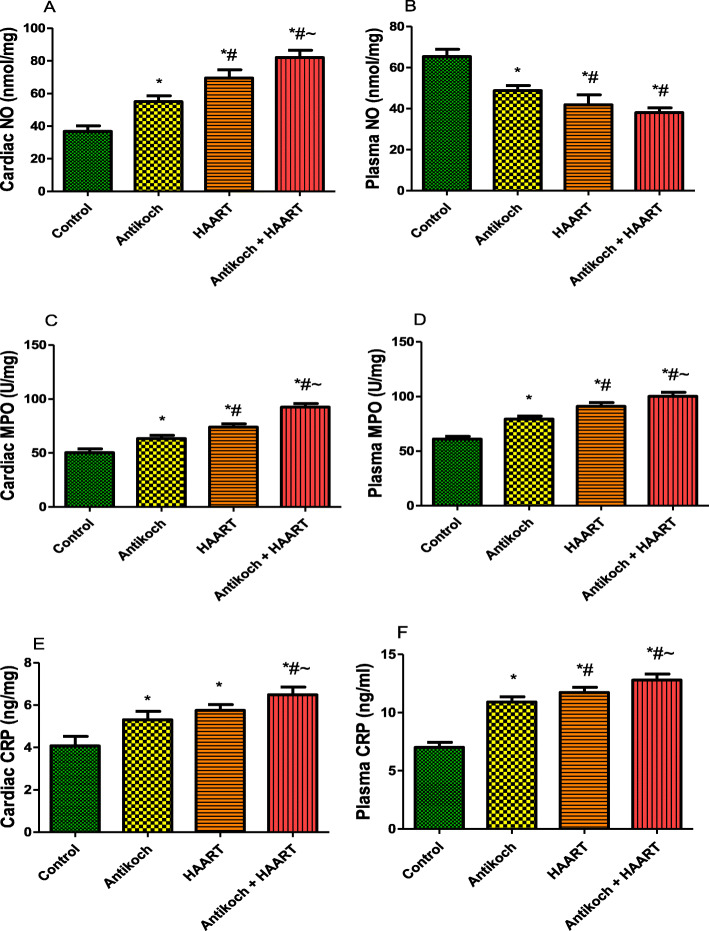
Effects of antikoch and HAART on cardiac and plasma nitric oxide (NO) levels (**A**, **B**), myeloperoxidase (MPO) activities (**C**, **D**), and C-reactive protein (CRP) levels (**E**, **F**).Data are presented as mean ± SD of 8 replicates; *P < 0.05 vs control; #*P <* 0.05 vs antikoch; ~*P <* 0.05 vs HAART

Treatment with antikoch and/or HAART significantly decreased plasma NO but increased cardiac NO content compared with the control. However, administration of antikoch and HAART, alone and concurrently significantly increased cardiac and plasma MPO activity and CRP levels compared with the vehicle-treated control (Fig. 8).

### Antikoch but not HAART alters cardiac cytoarchitecture

**Fig. 9:**
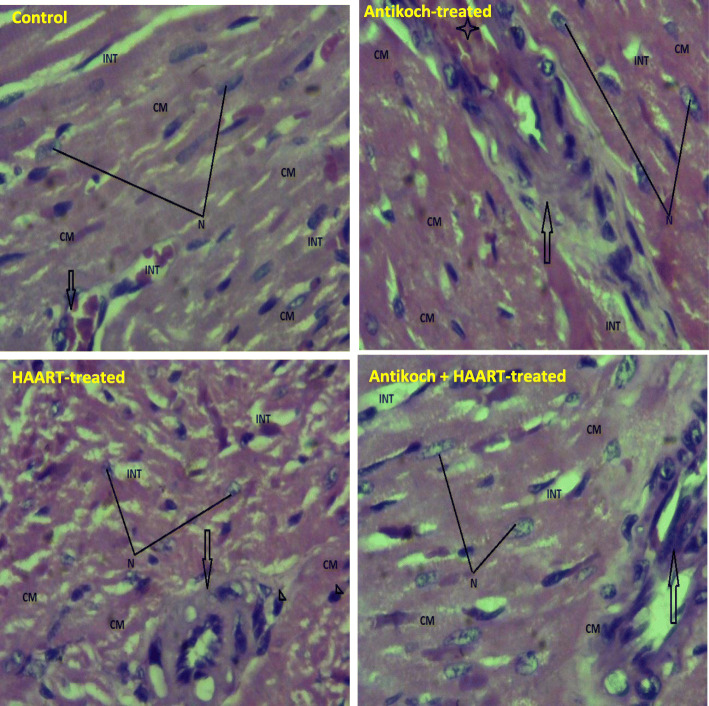
Effects of antikoch and HAART on cardiac cytoarchitecture Control: Photomicrograph shows the myocardium composed of the cardiac muscle (CM) consisting of myofibrils and well outline nucleus (N). The cardiac muscles are separated by the interstitium (INT) that is free from collection and congestion. The branch of the coronary vessels (Arrow) is well outlined and free from any form of microangiopathies. Section shows well preserved myocardia histoarchitecture. Antikoch-treated: Photomicrograph shows the myocardium composed of the cardiac muscle (CM) consisting of myofibrils and well outline nucleus (N). The cardiac muscles are separated by the interstitium (INT) that is free from collection and congestion. The branch of the coronary vessels (Arrow) is well outlined and appeared congested (star). Section shows well preserved myocardia histoarchitecture. HAART-treated: Photomicrograph shows the myocardium composed of the cardiac muscle (CM) consisting of myofibrils and well outline nucleus (N). The cardiac muscles are separated by the interstitium (INT) that is free from collection and congestion. The branch of the coronary vessels (Arrow) is well outlined and free from any form of microangiopathies. Section shows well preserved myocardia histoarchitecture. Antikoch + HAART – treated: Photomicrograph shows the myocardium composed of the cardiac muscle (CM) consisting of myofibrils and well outline nucleus (N). The cardiac muscles are separated by the interstitium (INT) that is free from collection and congestion. The branch of the coronary vessels (Arrow) is well outlined and free from any form of microangiopathies. Section shows well preserved myocardia histoarchitecture

Surprisingly, histopathological examination of the cardiac tissue revealed that similar to the control, administration of HAART alone or in combination with antikoch preserved the myocardia histoarchitecture. However, treatment with antikoch led to congestion of the coronary vessels (Fig. 9).

**Fig. 10:**
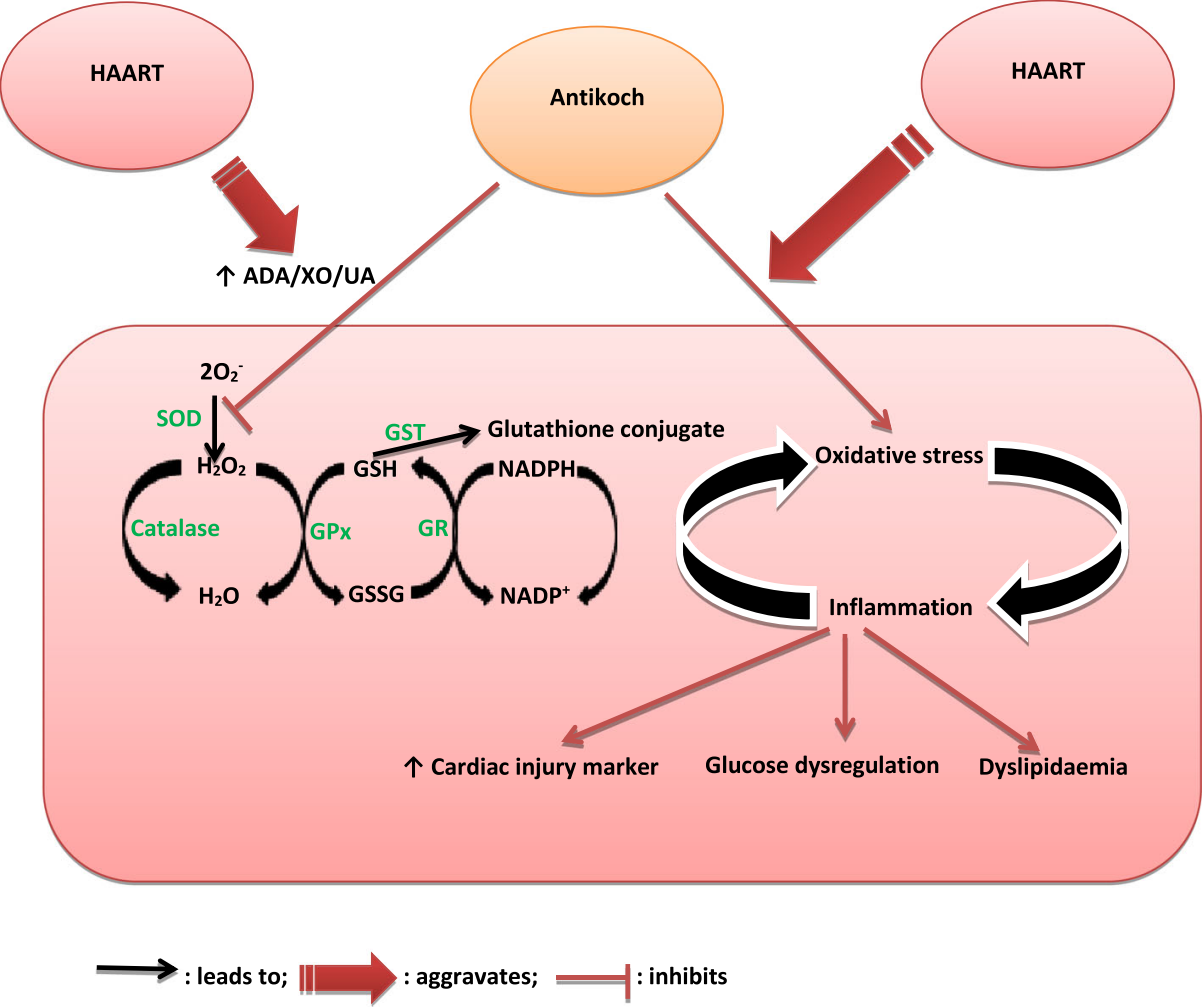
Schematic representation of the possible mechanism by which Antikoch and HAART induces cardiometabolic dysfunction. ADA: Adenosine deaminase, XO: xanthine oxidase, UA: uric acid, SOD: superoxide dismutase, GPx: glutathione peroxidase, GSH: reduced glutathione, GSSG: oxidized glutathione, GR: glutathione reductase, GST: glutathione-S-transferase, 2O_2_^-^: super oxide radical

## Discussion

The current study is seemingly the first to demonstrate the role of the glutathione system and ADA/XO/UA signaling in antikoch-and HAART-induced cardiometabolic dysfunction. Our findings showed that HAART exacerbates antikoch-induced insulin resistance, hyperglycaemia, and dyslipidaemia. These events were accompanied by elevated cardiac injury markers and atherogenic indices and associated with remarkable suppression of cardiac and plasma glutathione content and accumulation of ADA/XO-dependent UA in the plasma and cardiac tissue (Fig. 10).

Considerable evidence suggests that antikoch/HAART-induced cardiometabolic dysfunction results from complex activities on adipokines and the vascular system, especially the endothelial cell, which is a target of these drugs [43]. Our findings that antikoch and HAART blunted insulin sensitivity and induced hyperglycaemia as seen in type II diabetes, without an increase in body weight gain confirms the report of Noor et al. [44] among protease inhibitor users. This is likely due to inhibition of glucose transporter, GLUT4 [45]. GLUT4 is activated by insulin and promotes glucose uptake into the adipose tissues and muscle. The selective suppression of GLUT4 activity reported in the previous study of Noor et al. [45] explains, at least in part, the hyperglycaemia and insulin resistance observed in the present study.

Interestingly, the findings of the present open a new perspective to antikoch/HAART-induced cardiometabolic dysfunction. The present study showed that although antikoch/HAART depletes circulatory NO, these drugs led to a marked increase in cardiac NO content. NO plays a dual role. It is a potent vasodilator and acts through cGMP produced by the smooth muscle cells [28], thus acts as an anti-atherogenic factor by recusing platelet adhesion and aggregation. It may also interact with oxygen-derived radicals to generate cytotoxic molecules [28, 46]. Our findings on NO modulation corroborate earlier findings that protease inhibitors led to structural and functional vascular changes [47]. Depletion of circulatory NO enhances vascular smooth muscle cell proliferation and promotes the generation of the components of extracellular matrix with resultant increased arterial stiffness [48] and suppression of NO-mediated endothelial-dependent vasorelaxation [49]. This impairs cardiac perfusion and oxygenation and causes cardiac injury evident in the present study by elevated cardiac activities of CK and LDH and raised cardiac troponin and lactate. Although fatty acid oxidation is the preferential source of ATP generation in the myocardium, glucose oxidation is opted for in a pathological state [50]. Also, in anaerobic conditions, pyruvate is converted to lactate via the action of LDH. Antikoch/HAART-driven circulatory NO-depletion causing poor cardiac perfusion and oxygenation possibly switched ATP source to glucose oxidation with increased activity of LDH and lactate production to maintain myocardial energy balance and preserve cardiac cytoarchitecture.

The observed rise in cardiac NO and reduction in plasma NO as well as increased MPO activity (a marker of neutrophil infiltration and inflammation) and CRP levels (a marker of inflammation) in both plasma and cardiac tissue confirms that the vascular protective activity of NO is attenuated during inflammatory response secondary to the simultaneous generation of superoxide radical which reacts with NO to form peroxynitrite, a highly reactive species, that contributes to vascular injury [51]. Also, the generated peroxynitrite causes eNOS uncoupling, iNOS uncoupling, and depletion of tetrahydrobiopterin (BH_4_) [28]. This leads to increased peripheral resistance and impaired blood flow resulting in reduced cellular glucose uptake and utilization and ultimately insulin resistance [28, 52].

Furthermore, findings from the current study suggest UA as a key player in antikoch/HAART-induced cardiometabolic dysfunction. It could be inferred from our data that antikoch/HAART stimulated ADA/XO signaling which in turn enhanced UA generation. The generated UA induced NADPH oxidase (NOX) activity, production of reactive oxygen species (ROS), activation of mitogen-activated protein kinases (MAPK) p38, and extracellular signal-regulated kinases (ERK), a decrease in NO bioavailability, and raised protein nitrosylation and lipid oxidation [53]. These contribute to nitrosative/oxidative stress. Its reaction with peroxynitrite activates the pro-oxidant activity of UA; hence UA further depletes NO bioavailability, promotes inflammation and oxidative stress, and induces insulin resistance, glucose intolerance, dyslipidaemia, and impaired cardiovascular function [28–30]. NOX stimulates XO; both of which induce eNOS uncoupling and ROS generation [28]. It is possible that antikoch/HAART-induced elevated TG promotes the influx of accumulated LDL into the tunica intima where ROS oxidize it and macrophages pick it up through scavenger receptor (SR) CD36 to form foam cells [28, 54, 55]. Enhanced oxidative stress and inflammatory response enhance apoptosis of the foam cells and generation of necrotic lipid core contributing to the narrowing of arterial lumen and increased intraluminal pressure in atherosclerosis [28]. Also, the oxidized LDL is cytotoxic [28] and induces cardiotoxicity.

Our findings that antikoch/HAART-induce oxidative stress align with Lewis and his colleagues [56] that showed that nucleoside reverse transcriptase inhibitors inhibit DNA pol-γ which is responsible for the replication and maintenance of mtDNA. This leads to impaired mtDNA synthesis, depletion of functional mtDNA, and dysregulated mtRNA and mitochondrial protein synthesis and ultimately inhibits mitochondrial electron transport and oxidative phosphorylation. These events lead to reduced ATP generation, increased dependence on glycolysis, and further ROS generation [57]. This possibly accounts for the increased LDH and lactate production observed following antikoch/HAART exposure. Hence, it is likely that antikoch and HAART promote oxidative stress by activating multiple pathways, at least DNA pol-γ inhibition, and up-regulation of ADA/XO/UA signaling.

Notably, suppression of glutathione was observed to influence antikoch and HAART-induced cardiometabolic dysfunction. In the physiological state, the antioxidant defense system protects against ROS attack. SOD dismutates superoxide radicals to oxygen and hydrogen peroxide in the mitochondrial matrix [33, 58], thus eliminating superoxide and leaving hydrogen peroxide which also can induce injury [59]. Hydrogen peroxide is broken down to water via oxidation of glutathione by glutathione peroxidase [33, 60]. Glutathione reductase and NADPH recycle the oxidized glutathione [33, 61], while glutathione-S-transferase eliminates some as glutathione conjugates [33]. The observed antikoch/HAART-induced depletion of GSH and suppression of GPx and GST activities construes that the glutathione system was overwhelmed and thus increasing the susceptibility to ROS attack and penultimate oxidative stress.

## Study strength and limitations

The present study bridges the existing gap and avails scientific explanations on the impact of antikoch and HAART on cardiometabolic function. It also highlights the mechanistic role of the glutathione system and ADA/XO/UA pathway in antikoch/HAART-induced cardiometabolic dysfunction. Since this is a prospective study using an animal model, results of this study should be extrapolated to human with care.

## Conclusions

In summary, HAART exacerbates antikoch-induced glucolipid dysregulation and cardiac injury. Antikoch and HAART, when used singly or combined, led to ADA/XO/UA- dependent oxidative stress, inflammatory response, insulin resistance and hyperglycaemia, and dyslipidaemia. This infers that regular monitoring of glucose level, insulin sensitivity, lipid profile, and oxido-inflammatory markers is important in patients on antikoch and/or HAART for prompt diagnosis and management of cardiometabolic disorder if it ensues. Studies demonstrating the effects of enhancing glutathione content and downregulating ADA/XO/UA signaling during antikoch and/or HAART treatment are recommended. This will open novel therapeutic windows in the prevention of antikoch/HAART-induced cardiometabolic disorder.

## Data Availability

The data used for the study are available from the corresponding author upon request.

## Abbreviations

HAART: Highly active anti-retroviral therapy
TB: Tuberculosis
HIV: Human Immunodeficiency Virus
ADA: Adenine deaminase
XO: Xanthine oxidase
UA: Uric acid
NO: Nitric oxide
CRP: C-reactive protein
MDA: Malondialdehyde
MPO: Myeloperoxidase
GPx: Glutathione peroxidase
GST: Glutathione-S-transferase
GSH: Reduced glutathione
HDL-C: High-density lipoprotein
FBG: Fasting blood glucose
TG: Triglycerides
TC: Total cholesterol
LDL-C: Low-density lipoproteins
VLDL: Very low-density lipoproteins (VLDL)
non-HDL: Non- High-density lipoprotein
GLUT4: Glucose transporter 4
NIH: National Institute of Health
CK: Creatinine kinase
LDH: Lactate dehydrogenase
H&E: Hematoxylin and eosin
SD: Standard deviation.
ANOVA: One-way analysis of variance
cGMP: Cyclic guanosine monophosphate
ATP: Adenosine triphosphate
BH_4_: tetrahydrobiopterin
eNOS: Endothelial nitric oxide synthase
iNOS: Inducible nitric oxide synthase
NOX: NADPH oxidase
MAPK: Mitogen-activated protein kinases
ERK: Extracellular signal-regulated kinases
SR: Scavenger receptor
CD36: Cell differentiation 36
